# Impact of residues remote from the catalytic centre on enzyme catalysis of copper nitrite reductase

**DOI:** 10.1038/ncomms5395

**Published:** 2014-07-15

**Authors:** Nicole G. H. Leferink, Svetlana V. Antonyuk, Joseline A. Houwman, Nigel S. Scrutton, Robert R. Eady, S. Samar Hasnain

**Affiliations:** 1 Manchester Institute of Biotechnology, Faculty of Life Sciences, University of Manchester, Manchester M1 7DN, UK; 2 Molecular Biophysics Group, Institute of Integrative Biology, Faculty of Health and Life Sciences, Institute of Integrative Biology, University of Liverpool, Liverpool L69 7ZB, UK; 3 These authors contributed equally to this work

## Abstract

Enzyme mechanisms are often probed by structure-informed point mutations and measurement of their effects on enzymatic properties to test mechanistic hypotheses. In many cases, the challenge is to report on complex, often inter-linked elements of catalysis. Evidence for long-range effects on enzyme mechanism resulting from mutations remains sparse, limiting the design/redesign of synthetic catalysts in a predictable way. Here we show that improving the accessibility of the active site pocket of copper nitrite reductase by mutation of a surface-exposed phenylalanine residue (Phe306), located 12 Å away from the catalytic site type-2 Cu (T2Cu), profoundly affects intra-molecular electron transfer, substrate-binding and catalytic activity. Structures and kinetic studies provide an explanation for the lower affinity for the substrate and the alteration of the rate-limiting step in the reaction. Our results demonstrate that distant residues remote from the active site can have marked effects on enzyme catalysis, by driving mechanistic change through relatively minor structural perturbations.

## Abstract (web summary)

Residues within the catalytic site of enzymes are important for activity, but whether more distant residues are also sensitive to mutation is unclear. Here, Leferink *et al.* show that mutation of residues in copper nitrate reductase that are 12Å away from the active site perturb enzyme function.

## Graphical abstract

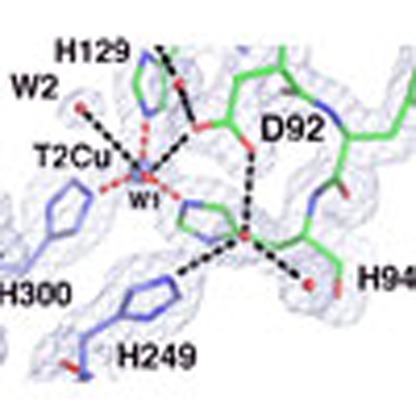

Enzyme catalysis of chemical reactions occur with high specificity and rates required by the biological process^1^,^2^. How these reactions are controlled and regulated^3^,^4^,^5^ has remained a major challenge to reproduce in synthetic enzymes as very often the focus has been on the catalytic core. The predictability of a mutation in a natural enzyme on enzymatic properties has improved tremendously over the last 20 years through the availability of high-resolution structural studies combined with enzymatic data. Despite this progress, our ability to predict essential structural elements of an enzyme for catalysis remains insufficiently advanced to be able to design/redesign a biological catalyst.

Copper-containing nitrite reductases (CuNiRs) catalyse the one-electron reduction of nitrite to nitric oxide: NO_2_^−^+2H^+^+e^−^ ↔ NO+H_2_O, a step in the microbial ATP-generating respiratory dentrification pathway^3^,^4^. It has recently been shown that protons, in addition to being a substrate in this reaction, are involved in controlling the rate of electron transfer (ET) between the type-1 Cu (T1Cu) centre and the catalytic type-2 Cu (T2Cu) centre of the enzyme^5^,^6^,^7^,^8^,^9^.

In common with many enzymes, targeted mutagenesis and extensive crystallographic studies of the CuNiR family have been powerful approaches to complement kinetic and theoretical studies to understand mechanism and function^10^,^11^,^12^,^13^,^14^. CuNiRs are trimeric and each monomer has two characteristic β-sandwich cupredoxin domains that contain a T1Cu centre with (Cys–Met–His_2_) ligation. The active site is a T2Cu with (His_3_–H_2_O) ligation, which is situated at the interface between two adjacent monomers in a cleft ~12 Å from the protein surface. Electrons produced during denitrification are donated to the T1Cu site by a partner redox protein^15^ and subsequently transferred via a 12.6-Å His–Cys bridge to the T2Cu centre in a proton-gated reaction^5^,^7^. Access to the T2Cu centre is by way of a ~6-Å-wide hydrophobic channel formed by residues from adjacent monomers of the trimer. The active site pocket around the T2Cu centre has an Asp and a His residue conserved in all CuNiRs. These residues are essential for effective catalysis and are proposed to act in acid base catalysis to provide the two protons required for nitrite reduction^16^,^17^,^18^, and the rate-limiting catalytic step involves a single protonation event kinetically linked to ET^5^. In the oxidized enzyme as isolated, the Asp residue is hydrogen bonded to a water molecule bound to the T2Cu. This liganded water at the T2Cu is displaced when nitrite binds. Reduction of the T2Cu in the absence of nitrite can result in dissociation of this water to form a three coordinate site incapable of binding substrate^14^,^19^. Inactivation is prevented if nitrite displaces water from the reduced T2Cu before it can dissociate. Thus, during enzyme turnover in the presence of excess reductant, the delivery of electrons to the active site has to be finely tuned to the availability and binding of substrate.

Altering access from bulk solvent to a buried catalytic centre by substituting residues within the access channel has been shown to change the reaction mechanism. Here we show that substitution of a surface residue (F306C) at the entrance of the channel of CuNiR results in an altered second sphere coordination of the active site Cu. This changes the rate-limiting step resulting in an increase in inter-molecular ET and catalytic activity despite a significantly lower affinity for substrate.

## Results

### Structure of the mutant and its substrate-bound form

We mutated Phe306, a surface residue at the mouth of the proton/substrate access channel of *Ax*CuNiR, determined its crystallographic structure and used laser-flash photo-excitation methods to rapidly transfer electrons to the T1Cu site to measure rates of intra-molecular ET, the activity profile and the nitrite concentration dependence of these processes.

**Figure 1:**
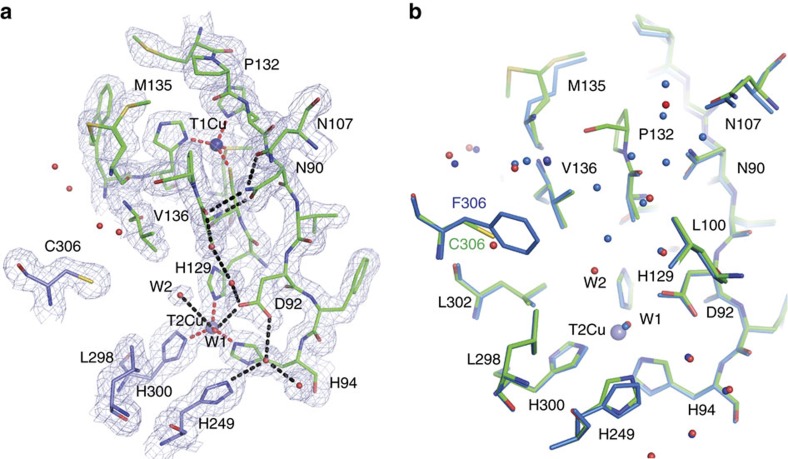
Comparison of the main proton channel network in as-isolated F306C mutant and wild-type *Ax*NiR. (**a**) The main proton channel of F306C has an additional water molecule, W2, that forms an hydrogen bond with the liganded water of the T2Cu ion. The 2*F*_o_–*F*_c_ electron density map for F306C mutant contoured at the 1σ level. Distances from Cu atoms to coordinating protein atoms are shown as dotted red lines. Hydrogen bonds are shown in black dotted lines. Water molecules are shown as red spheres and Cu atoms as blue spheres. Residues are colour coded according to their chain. (**b**) Comparison between wild-type *Ax*NiR (blue) and F306C mutant (green). Water molecules for the mutant are shown in red, whereas for wild-type protein in blue.

**Figure 2:**
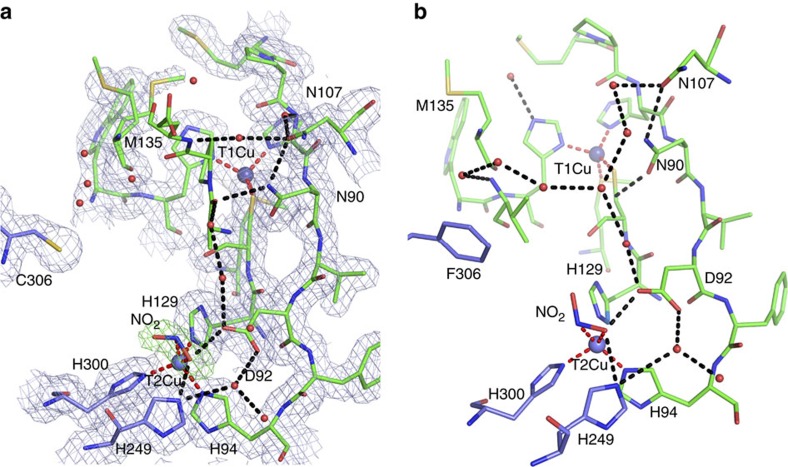
Comparison of the nitrite-binding mode in as isolated F306C mutant and wild-type *Ax*NiR. 2*F*_o_–*F*_c_ electron density maps contoured at 1σ level for both (**a**) as-isolated F306C mutant and (**b**) wild-type AxNiR. The Cu sites are shown as blue spheres with coordination bonds shown as dashed red lines. Important hydrogen bonds are shown as dashed black lines. 1*F*_o_–*F*_c_ omit map for nitrite is shown in green at 4σ level. Note that crystals of F306C were grown in the presence of PEG that prevents nitrite diffusion into crystals of wild-type A*x*NiR^10^, consistent with the mutation resulting in enhanced access to the active site.

**Figure 3:**
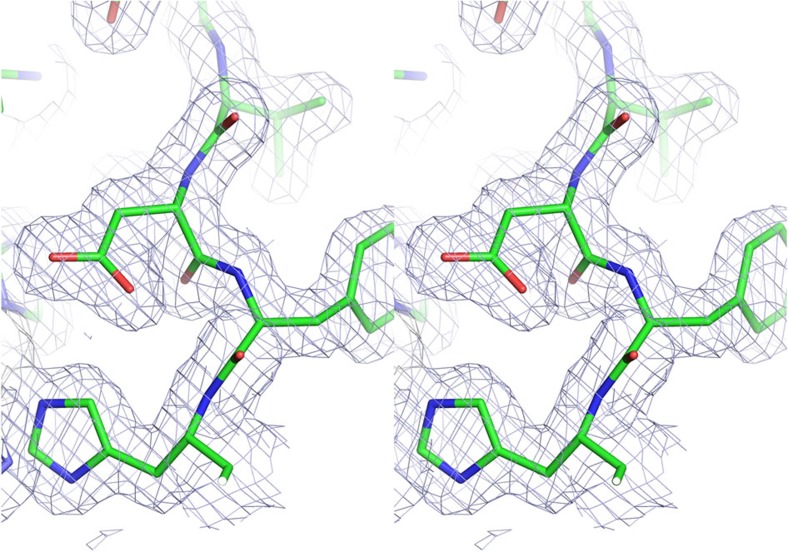
A stereo picture of the 2F_o_–F_c_ electron density map. Electron density at 1σ level around His94, Phe93 and Asp92 for the structure of the resting state of F306C mutant.

Comparison of the crystallographic structures of the ‘as isolated’ (1.7 Å resolution) and substrate bound (1.75 Å resolution), (Table 1) allowed us to assess the effect of this substitution on the substrate access channel and its associated water network. The global structure of the F306C enzyme was very similar to the wild-type *Ax*NiR (2vw4) (ref. 10) with an average C_α_ atom root mean squared deviation of 0.20 Å and maximum displacement 1.5 Å in the N terminus region. The details of the substrate access channel and comparison with wild type is shown in Fig. 1a,b. The substitution did not change the highly ordered hydrogen bonded water network (Asp92–water–water–Ala131–Asn90–Asn107) from the T2Cu site to the protein surface^20^ that provides the major route of proton delivery to the active site. A notable feature of the channel was the presence of an additional water molecule hydrogen bonded to the liganded water of the T2Cu ion. This second water molecule has no counterpart in the numerous structures of other two domain CuNiRs, and presents an altered energy landscape for nitrite displacement of the Cu-coordinated water molecule during catalysis. Access to the active site from bulk solvent was also improved, as illustrated by the ability of nitrite to diffuse into the T2Cu active site in the crystal despite polyethylene glycol molecule (PEG) (present as a component of the crystallization medium) being bound across the mouth of the substrate channel. Previously, we have shown that the T2 site is not accessible to nitrite in crystals of wild-type *Ax*NiR where PEG effectively blocks the channel^10^. Although there is some variation in the positioning of nitrite at the T2 site in CuNiRs, our structure shows that nitrite is bound in a similar mode to wild-type *Ax*NiR (2xwz) (ref. 7). The details of nitrite binding are shown in Fig. 2. The space generated by the F306C mutation also allows an adjacent surface residue at the mouth of the channel, Met135, to adopt a dual conformation. The binding of nitrite to the T2Cu results in Met135 adopting largely an ‘open’ conformation across the substrate access channel, suggesting a communication linkage between the substrate-bound T2Cu and the distant Met135 (Fig. 2). In fact, this change has been observed in the wild-type *Ax*NiR enzyme and several other CuNiRs upon nitrite binding but its significance had remained unnoticed. The sensor loop^10^ (His94-Asp92-His89) that reports the status of the T2Cu site to the electron-donating T1Cu site remains unaltered. An example of a stereo electron density involving His94-Asp92 is shown in Fig. 3.

### Catalytic activity of the mutant

Steady-state nitrite reductase activity of F306C was measured by NO formation by using our recently developed assay^5^ that removes potential ambiguity arising from the reversibility of NiR, and also the side reaction of the reduction of the product NO in the bulk solvent to form N_2_O. Both wild-type *Ax*NiR and the F306C variant followed normal Michaelis–Menten kinetics with nitrite concentrations up to 50 mM, suggestive of a single site of nitrite binding. However, the substitution of F306 resulted in an approximately fourfold increase in specific activity and had a marked effect on the apparent *K*_M_ for nitrite, which with a value of 1.2±0.1 mM is some 50-fold weaker than for wild-type *Ax*NiR. Kinetic parameters are summarized in Table 2. Since the structure shows access to the substrate channel is enhanced, the weaker apparent affinity for nitrite may arise from the presence of the additional water in the second coordination sphere of the T2Cu stabilizing the coordinated water that nitrite needs to displace in order to bind. An additional contributing feature revealed by the structure is the positioning of the gatekeeper Asp92 residue in the ‘closed’ conformation^11^ that may result in a slower escape of NO from the active site pocket, resulting in a weaker apparent *K*_M_ value for nitrite due to competitive binding of NO to T2Cu in the micro-reversibility of NiR function.

**Figure 4:**
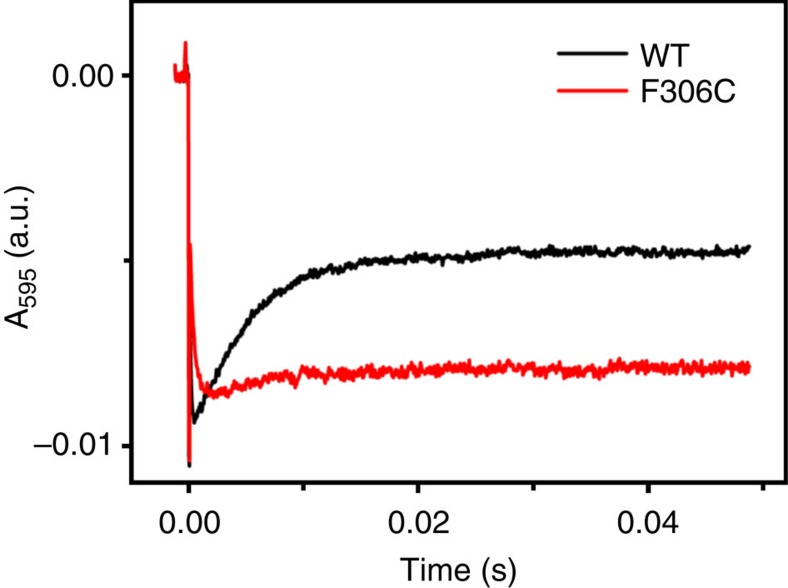
Time-resolved absorbance changes upon laser excitation at pH 5.8 in the absence of nitrite. Absorbance changes at 595 nm observed in the laser flash-photolysis instrument during reduction of wild-type (WT) *Ax*NiR (black) and F306C (red) with NADH (*λ*_exc_=355 nm). Experiments were carried out in the absence of nitrite in MMH buffer, pH 5.8, containing 30 μM WT *Ax*NiR or F306C, NiR, 200 μM NADH and 50 mM mediator at 4 °C. Traces shown are the average of 3–6 individual traces and were fitted by using a double-exponential equation. Observed rates of inter-Cu ET for WT *Ax*NiR and F306C are 200 and 160 s^−1^, respectively. The lower absorbance at equilibrium observed for F306C is consistent with an ~60 mV decrease in the *E*_m_ of the T2Cu centre.

The kinetics of inter-Cu ET from T1Cu to T2Cu was measured by using laser-flash photo-excitation methods to rapidly transfer electrons to the T1Cu site as described previously^5^,^6^ (Fig. 4). Following the initial rapid reduction of T1Cu, an electron redistributes between the two Cu centres as the thermodynamic redox equilibrium is re-established. The effect of the F306C substitution was to slow this ET reaction from 370 s^−1^, seen in wild-type *Ax*NiR^5^, to ~170 s^−1^. Since there is no significant change to the redox potentials of the Cu centres at this pH (Table 3) the slower rate most likely arises from an increase in reorganizational energy associated with the altered water structure of the T2Cu sites.

Several pulse radiolysis^16^,^21^,^22^, protein-film voltammetry^9^,^19^ and laser-flash-photolysis studies^5^,^7^ of different CuNiRs have shown that during single turnover experiments, the rate of inter-Cu ET is decreased by the presence of nitrite. The degree of inhibition is pH-dependent and for wild-type *Ax*NiR at pH 7 the *k*_obs_ decreases from 370 to ~250 s^−1^ at saturating nitrite concentrations^5^,^7^. The effect of nitrite in inhibiting the rate of ET has been suggested to arise from conformational and midpoint potential changes of the T2Cu centre on replacement of water by nitrite^23^. This seems counter-intuitive to the observed rise in the *E*_m_ of the T2Cu centre on binding nitrite, thus increasing the driving force for ET, and more likely is due to a rate-limiting proton-gating event associated with catalysis. The nitrite concentration dependence of the inhibition of ET showed an initial inhibition followed by an increase at higher concentrations. This has been attributed^5^,^7^ to binding at two sites with different affinities or complexities arising from reaction branching associated with the random sequential mechanism for NiR function^5^,^23^. Whatever the origin of this complex behaviour, the different substrate dependences of internal ET and the steady-state activity show that inter-Cu ET cannot be rate limiting in wild-type *Ax*NiR at pH 7.

### Effect of nitrite concentration on the rate of ET

**Figure 5:**
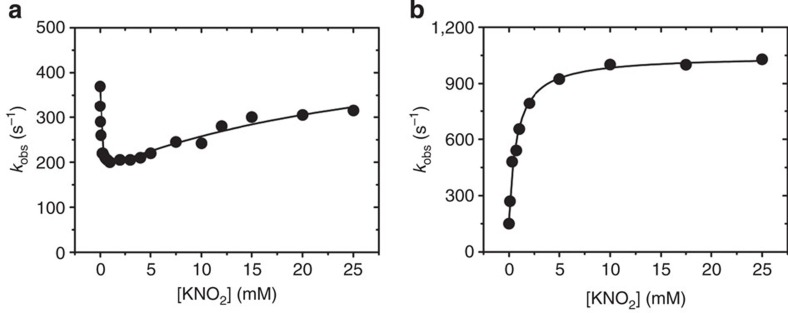
Substrate dependence of inter-Cu ET in laser photo-excitation experiments. Wild-type *Ax*NiR (**a**) and F306C (**b**). All experiments were carried out in 50 mM potassium phosphate buffer, pH 7.0, at 4 °C. The solid line in **a** represents a fit of the data obtained for wild-type *Ax*NiR to an equation reflecting two substrate-binding sites, and in **b** a fit of the data obtained for F306C to a hyperbolic one-site binding equation. The data shown in **a** contains re-plotted data from ref. 5.

A marked difference in behaviour of F306C was observed in the effect of nitrite concentration on the rate of ET. Although starting from a lower value under non-turnover conditions, inter-Cu ET was not inhibited by nitrite but stimulated, showing a ‘normal’ hyperbolic saturation dependence, in contrast to the complex behaviour of the wild-type enzyme (Fig. 5a,b). At saturating nitrite concentrations, the *k*_obs_ of ~920 s^−1^ was some fourfold faster than for wild-type *Ax*NiR^5^, and is associated with an approximately fourfold increase in enzymatic activity. The apparent affinity for nitrite obtained from the laser flash-photolysis data (*K*_s_=0.8±0.1 mM) is in the same range as the apparent *K*_M_ value obtained from the steady-state activity (*K*_M_=1.2±0.1 mM). These data, together with the similar patterns of nitrite dependence of activity in steady-state assays and inter-Cu ET, clearly show that ET has now become the rate-limiting step in turnover of F306C at pH 7, and results in a single site of nitrite binding.

**Figure 6:**
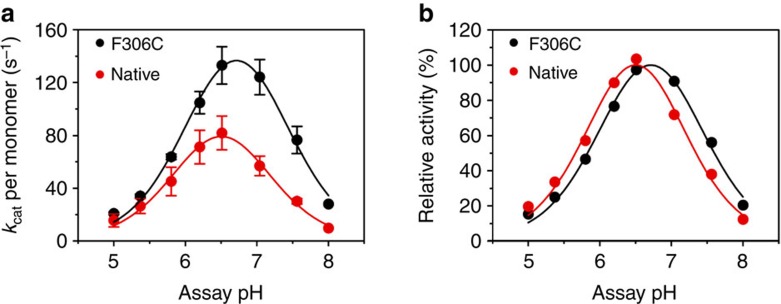
pH dependence of nitrite reductase activity of F306C compared with wild-type *Ax*NiR. Steady-state activities of NiR activity were measured by using dithionite as electron donor. The oxidation of dithionite was measured spectrophotometrically at 315 nm (*?*_315_=8.043 mM^−1^ cm^−1^) in a stopped-flow apparatus by using MMH buffer, titrated to the indicated pH, at 4 °C (refs 5, 8). Initial rates were determined from five averaged progress curves and corrected for the background depletion of absorbance at 315 nm in the absence of *Ax*NiR. Data shown are the mean±s.d. of two individual experiments. The activity profile for the wild-type enzyme is different from that published by Abraham *et al.*^34^, which was measured by using a stopped-time assay. The optimum for wild-type *Ax*NiR found in the current study is ~pH 6.5, rather than pH 5.2, also no plateau at pH 5.8–6.2 is observed. This ‘new’ optimum pH is similar to the pH optimum of 6.2 found for *Ac*NiR and *Af*NiR. The pH optimum for F306C is slightly shifted to pH 6.7 compared with the wild-type enzyme. (**a**) Observed rates versus pH, and (**b**) relative activity versus pH. The pH dependence of the kinetic parameters were fitted to an equation describing a double ionization^35^. The calculated macroscopic p*K*_a_ values are 6.0 and 7.0 for wild-type *Ax*NiR, and 6.1 and 7.3 for F306C.

These changes are not due to an effect on the midpoint potentials since the difference in redox potential between the T1Cu and T2Cu centres provides little driving force for ET at pH 7 or 5.8 (Table 3). It most likely arises from perturbation of a protonation event coupled to substrate reduction rather than inter-Cu ET, since in the absence of nitrite this is slower in the mutant. Recent computational data indicate that in CuNiRs, ET is triggered by the increase in potential of the reduced T2 site when nitrite binds, gated by the protonation of the conserved Asp residue in the active site pocket^6^. The pH dependence of ET in the absence of nitrite reveals that the F306C mutation abolishes proton gating (Fig. 4). The rates observed at pH 5.8 and 7 are almost identical in F306C, 160 and 170 s^−1^ respectively, compared with a significantly slower inter-Cu ET at pH 5.8 relative to pH 7 for wild-type *Ax*NiR, 200 and 370 s^−1^, respectively (Table 4). This is confirmed by the altered pH profile of the activity of the mutant, which shows optimal activity at pH ~6.7, and a somewhat broader maximum than the wild-type enzyme, which shows maximal activity at pH ~6.5 (Fig. 6).

## Discussion

We have shown previously that perturbation of the main proton channel in the N90S substitution of *Ax*NiR has a similar effect on the nitrite concentration dependence of ET to the F306C substitution^7^. However, although an additional water molecule was present in the substrate access channel, it was not hydrogen bonded to the coordinated water at the T2Cu site. The effects of surface residue substitution, F306, described here produce sufficient flexibility to allow increased solvent/substrate access, illustrated by the presence of a second water molecule (W2) in the active site pocket and also improved access to nitrite since binding is not prevented by PEG bound at the channel’s entrance. Because access of both water and nitrite is enhanced, we attribute the 50-fold increase in apparent *K*_M_ for nitrite not to there being more water to compete with, but rather arising from stabilization of the ligated water owing to its hydrogen bonding to W2, making it more difficult for nitrite to displace. In native CuNiR, access of water to the active site pocket is not limited by surface residues blocking the channel but rather by steric hindrance provided by an isoleucine residue shielding the active site^24^. Several computational studies have shown the importance of second sphere ligands in modulating the energy landscape of the T2Cu site, specifically the effect of the position and protonation state of the ligated water molecule^25^.

The decrease in catalytic efficiency (*k*_cat_/*K*_M_) shows the importance of a bulky residue at the surface of the substrate channel in restricting solvent access to the active site pocket in the native enzyme. The overall tight regulation of the reaction chemistry in terms of coordinated electron and proton supply to, and nitrite availability at, the active site of CuNiRs prevents the formation of a deactivated enzyme species through premature reduction of T2Cu leading to loss of the coordinated water ligand. It is clear that this fine-tuning of the reaction chemistry extends to surface residues remote from the active site. This result has wider implications to the predictive aspects of design/redesign of synthetic biological catalysts that are of central importance to industrial biocatalysis.

## Methods

### Cloning and mutagenesis

The *nirA* gene from *Alcaligenes xylosoxidans* (*Ax*NiR) was re-cloned for cytoplasmic expression to improve expression levels. *NirA* without its periplasmic signal peptide was PCR amplified from a vector containing the wild-type *nirA* gene^26^ by using the oligonucleotide primers *Ax*NiR-SPfw (5′-CCCGTCTCCCATGCAGGACGCCGACAAGC-3′), *Ax*NiR_rv (5′-GGAAGCTTTCAGCGCGGAATCGGC-3′) (restriction sites are underlined and changed nucleotides are in bold), and cloned between the *Nco*I and *Hin*dIII restriction sites of the pET28a vector (Novagen, San Diego, CA, USA), resulting in the plasmid pET-*Ax*NiR-SP. Site-directed mutagenesis to generate F306C was performed by using pET-*Ax*NiR-SP as template and using the oligonucleotide primers F306C_fw (5′-CCACAACCTGATCGAGGCCTGCGAACTGGGCGCGGCCGGCCAC-3′) and F306C_rv (5′-GTGGCCGGCCGCGCCCAGTTCGCAGGCCTCGATCAGGTTGTGG-3′) according to the QuikChange protocol (Stratagene, La Jolla, CA, USA). Mutagenesis was confirmed by automated sequencing (Eurofins MWG Operon, London, UK).

### Protein expression and purification

*Escherichia coli* BL21(DE3) cells harbouring a pET-*Ax*NiR-SP plasmid were grown in 1 litre cultures of Luria–Bertani medium supplemented with 50 μg ml^−1^ kanamycin at 37 °C until an OD_600_ of ~0.6 was reached. Protein expression was induced by the addition of 0.5 mM (isopropyl-D-1-thiogalactopyranoside) IPTG and 1 mM CuSO_4_. Incubation was continued for 16 h before harvesting by centrifugation. The cell pellet was re-suspended in 20 mM MES, pH 6.0, and the cells were broken by ultra-sonication. The lysate was cleared by centrifugation and dialysed against 20 mM MES and 1 mM CuSO_4_, pH 6.0, to restore the Cu content of the T2Cu centre. This was followed by further dialysis against deionized water (dH_2_O). The cell extract was then applied onto a carboxymethyl–cellulose (Whatman, Kent, UK) column equilibrated with dH_2_O. The column was washed with 10 column volumes of dH_2_O, 10 column volumes of 40 mM MES, pH 6.0, and 10 column volumes of 40 mM MES and 50 mM NaCl, pH 6.0. The protein was eluted with 300 mM NaCl in 40 mM MES, pH 6.0, and stored at −80 °C in 20 mM Tris, pH 7.4, until further use. *Ax*NiR concentrations were determined by using an extinction coefficient of *?*_595_=6.3 mM^−1^ cm^−1^ for both the wild-type enzyme as well as F306C.

### Structure determination

Crystals for the F306C and F306C+NO_2_^−^ structures of *Ax*NiR were grown by using the hanging drop vapour diffusion method at room temperature. Protein solution, 2 μl of 10 mg ml^−1^, in 20 mM Tris-HCl (pH 7.4) was mixed with an equal volume of reservoir solution containing 15% PEG550 MME, 50 mM ZnSO_4_, and 50 mM MES buffer (pH 6.5). Crystals grew within 2 days. Before being frozen in liquid nitrogen crystals were transferred into 30% PEG550 MME, 50 mM ZnSO_4_ and 100 mM MES buffer, pH 6.5, for 10 s nitrite-bound F306C was obtained by soaking crystals in 200 mM NaNO_2_^−^, 20% PEG550 MME, 50 mM ZnSO_4_ and 50 mM MES buffer for 15 min before flash cooling in 200 mM NaNO_2_^−^, 25% PEG550 MME, 50 mM ZnSO_4_ and 50 mM MES buffer. Crystallographic data were collected on Soleil beamline PROXIMA1 by using a PILATUS-6M detector to 1.69 Å resolution for F306C, and on DIAMOND’s microfocus beamline ID-24 to 1.75A resolution for the F306C+NO_2_^−^ complex. Data for as-isolated F306 structure were integrated and scaled by using XDS^27^. Data for F306C+NO_2_^−^ complex were integrated in MOSFLM^28^ and scaled by Scala^29^. The structure of mutant was refined starting from isomorphous model of the wild-type structure (2vw4), whereas F306C+NO_2_^−^ model was refined starting from isomorphous model 4csp. Both structures were refined by using REFMAC^30^ in the CCP4 (ref. 31) programme suite with isotropic B-factors and hydrogen atoms in riding positions. Rebuilding of the model between refinement cycles and adding water molecules was performed in Coot^32^. The quality of the models was assessed by using the Molprobity^33^ server. A summary of diffraction data, refinement statistics and the quality indicators for the structures are given in Table 1.

### Kinetic experiments

Protein samples for all kinetic experiments were prepared and handled anaerobically in a glovebox (Belle Technology, Portesham, UK) under a nitrogen atmosphere in which oxygen levels were kept below 5 p.p.m. The steady-state NiR activity was measured in a stopped-flow instrument (Applied Photophysics SC18MV) by using deoxyhemoglobin as NO scavenger^5^. All measurements were carried out in 50 mM potassium phosphate buffer, pH 7.0, at 4 °C. Absorbance changes were recorded at 430 nm, and initial rates were determined from the slopes of five averaged progress curves and converted to units of activity by dividing through a calibration factor of 0.0162 ΔA430 μM^−1^ (ref. 7). The steady-state activities of native *Ax*NiR and F306C as a function of pH were measured by using dithionite as electron donor. The oxidation of dithionite was measured spectrophotometrically at 315 nm (*?*_315_=8.043 mM^−1^ cm^−1^) in the stopped-flow apparatus by using 25 mM MES–25 mM HEPES–25 mM maleic acid (MMH), titrated to the indicated pH, at 4 °C (refs 5, 8). Initial rates were determined from five averaged progress curves and corrected for the background depletion of absorbance at 315 nm in the absence of *Ax*NiR.

Laser flash-photolysis experiments were carried out on solutions containing 30 μM NiR, 200 μM NADH and 50 mM *N*-methyl nicotinamide, and when appropriate, various concentrations of KNO_2_^−^ in 50 mM potassium phosphate buffer, pH 7.0 or 25 mM MMH buffer, pH 5.8. All laser experiments were performed at 4 °C. The samples were excited at 355 nm by using the third harmonic of a Q-switched Nd:YAG laser (Brilliant B, Quantel). Spectral transients were collected at 595 nm for up to 50 ms by using an Applied Photophysics LKS-60 flash-photolysis instrument, and fitted to a double-exponential equation.

### Reduction potentials

The reduction potentials of T1Cu and T2Cu were determined by electrochemical titration. Protein solutions (33 μM *Ax*NiR, equivalent to 100 μM T2Cu) were titrated with sodium dithionite as the reductant. Mediators (phenazine methosulphate (2 μM), methyl viologen (0.5 μM) and benzyl viologen (1 μM)) were added to facilitate electrical communication between enzyme and electrode before titration. The electrode potential was allowed to stabilize between each addition. The optical absorbance at 595 nm of oxidized T1Cu was monitored at each potential by using a Cary UV-50 Bio UV–visible scanning spectrophotometer. The electrochemical potential of the solution was measured by using a Thermo Orion ORP electrode at 25 °C. A factor of 210 mV was used to correct values to the standard hydrogen electrode. During the titration with dithionite, samples (250 μl) were withdrawn for EPR analysis. The samples were placed in 4 mm Suprasil quartz EPR tubes (Wilmad LabGlass) and sealed inside the glovebox where they were immediately removed and frozen in liquid nitrogen. Samples were stored in liquid nitrogen to prevent re-oxidation until they were analysed. EPR spectra were recorded at 20 K on a Bruker EMX X-band EPR spectrometer. The microwave power was 0.5 mW, the modulation frequency 100 kHz, and the modulation amplitude 5 G. The T1Cu reduction potentials were determined from the difference in absorbance at 595 nm between oxidized and reduced T1Cu, and the T2Cu reduction potentials were determined from the difference in the hyperfine peaks of the EPR spectrum between oxidized and reduced T2Cu. Midpoint potentials were obtained from fitting the data to the Nernst equation. Because the T1Cu reduction potential is pH independent^16^, the reduction potentials for T2Cu at pH 5.8 were calculated from the relative absorbance recovery observed in the flash-photolysis experiments at pH 5.8 by using the Nernst equation.

## Author contributions

S.S.H., R.R.E. and N.S.S. conceived and designed the project; J.A.H. cloned the mutant, N.G.H.L. purified proteins and performed kinetic experiments; S.V.A. crystallized and undertook X-ray data collection, processing, structure determination and refinement; N.G.H.L., S.V.A., R.R.E., N.S.S. and S.S.H. wrote the manuscript.

## Additional information

**How to cite this article:** Leferink, N. G. H. *et al.* Impact of residues remote from the catalytic centre on enzyme catalysis of copper nitrite reductase. *Nat. Commun.* 5:4395 doi: 10.1038/ncomms5395 (2014).

**Acceession codes:** Atomic coordinates and structure factors for the reported crystal structures have been deposited in the Protein Data Bank under accession codes 4csp(r4cspsf) and 4csz(r4cszsf).

## Figures and Tables

**Table 1:** Data collection and refinement statistics*.

	**F306C**	**F306C+NO**_**2**_
*Data collection*		
Space group	R3	R3
* *Cell dimensions		
*a*, *b*, *c* (Å)	89.9, 89.9, 289.5	89.6, 89.6, 144.0
*α*,*β*,*γ* (°)	90, 90, 120	90, 90, 120
Resolution (Å)	50–1.69 (1.79–1.69)	40–1.75 (1.84–1.75)
*R*_merge_	7.0 (80.0)	8.6 (66.8)
*I*/σ*I*	11 (2)	10 (2)
Completeness (%)	100 (99)	99.9 (100.0)
Redundancy	5 (5)	4.5 (4.5)
*Refinement*		
Resolution (Å)	46.5–1.7	40–1.75
No. of reflections	97,508	43,456
*R*_work/_*R*_free_	18.7 (22.4)	17.9 (21.0)
No. of atoms	5,850	2,959
Protein	5,074	2,617
Ligand/ion	36	19
Water	740	323
* B-factors*		
Protein	24.6	28.64
Cu	20	23.5
Zn	30	41
MES	47	
NO_2_		40
PEG		54
Water	35.2	37.8
*R.m.s.d.*		
Bond lengths (Å)	0.015	0.012
Bond angles (°)	1.616	1.542
PDB access codes	4csp	4csz

NO_2_, nitrite; r.m.s.d., root mean square deviation.

^*^(F306C -no Ramachandran outliers, for 660 residues 97.92% favoured 2.08% allowed; F306C+NO_2_—no Ramachandran outliers, for 331 residues 96.78% favoured, 3.22% allowed).

**Table 2:** Steady-state kinetic parameters of F306C compared with wild-type *Ax*NiR.

	***k***_**cat**_ **(s**^**−1**^)	***K***_**M**_ **NO**_**2**_^**−**^ **(mM)**	***k***_**cat**_**/*****K***_**M**_ **(mM**^**−1**^ **s**^**−1**^)
Wild-type *Ax*NiR*	89±3	0.027±0.005	3,300
F306C	310±10	1.2±0.1	260

NO_2_, nitrite.

^*^Data obtained from ref. 7. Values shown are the mean±s.d. of two individual experiments. The apparent kinetic constants for both the wild-type and F306C summarized here were obtained by nonlinear fitting of the data.

**Table 3:** Reduction potentials for T1Cu and T2Cu centres in wild-type *Ax*NiR and F306C.

	**T1Cu (mV)**	**T2Cu (mV)pH 7**	**T2Cu (mV)pH 5.8**
WT *Ax*NiR	255±3*	244±18*	260±15
F306C	262±3	269±10	210±15

NO_2_, nitrite; r.m.s.d., root mean square deviation; T1Cu, type-1 Cu; T2Cu, type-2 Cu; WT, wild type.

^*^Values are taken from ref. 7. Values shown for T1Cu and T2Cu at pH 7 are obtained after fitting the data sets from a single experiment to the Nernst equation. Since the T1Cu reduction potential is pH independent^16^, the values for T2Cu at pH 5.8 were calculated from the relative absorbance recovery in the flash-photolysis experiments performed at pH 5.8.

**Table 4:** Rates of inter-Cu electron transfer in wild-type *Ax*NiR and F306C in the absence of nitrite at pH 5.8 and 7.0.

	**pH 7.0 (s**^**−1**^)	**pH 5.8 (s**^**−1**^)
WT *Ax*NiR	370*	200
F306C	170	160

WT, wild type.

^*^Value are taken from ref.^5^. Values obtained are an average of 3–6 shots.
